# International research to address the challenges of metastatic breast cancer: the AURORA Program (BIG 14-01)

**DOI:** 10.1038/s41523-023-00548-9

**Published:** 2023-05-23

**Authors:** Carmela Caballero, Alexandre Irrthum, Theodora Goulioti, David Cameron, Larry Norton, Martine Piccart

**Affiliations:** 1 Breast International Group, Brussels, Belgium; 2 Cancer Research UK Edinburgh Centre, Edinburgh, UK; 3 Memorial Sloan Kettering Cancer Center, New York, USA; 4 Institut Jules Bordet, Brussels, Belgium

**Keywords:** Breast cancer, Metastasis, Predictive markers

## Abstract

Despite recent advances in breast cancer research, we still know little about the mechanisms that lead to metastatic breast cancer (MBC). However, treatment options for patients have increased based on results of recent randomized clinical trials in this setting. Today we have much hope, yet many questions remain unanswered. Conducting a fully academic and international study such as AURORA is very challenging, yet ever more crucial to advancing knowledge about MBC.

## Challenges in metastatic breast cancer research

What does it mean for a patient to be diagnosed with MBC today? It means being diagnosed with a cancer that is incurable. It means being subjected to multiple treatments with a high burden of side effects and frequent hospital visits, without having a definitive prognosis. It means having too little time to be with family and friends, and death can be near.

Despite recent advances, we still know little about the mechanisms that lead to MBC. However, treatment options for patients are increasing due to relentless efforts to understand this disease. Recent trials demonstrated statistically significant and clinically meaningful benefits in improving outcomes for both metastatic hormone-positive and HER2-positive breast cancer^1–3^. These unprecedented magnitudes of benefit from randomized clinical trials in the setting of MBC give us much hope even as new questions emerge.

What we know is that breast cancer is biologically complex and that the assessment of biomarkers beyond hormone receptors (ER, PR) and HER2 is essential for treatment decisions in the metastatic setting^4,5^. We know more about how breast cancer evolves. A first analysis of the AURORA program shows that alterations in several driver genes were enriched in metastatic lesions, including ESR1, PTEN, CDH1, PIK3CA and RB1 mutations, MDM4 and MYC amplifications, and ARID1A deletions. Clonality was also enriched in genes like ERBB2 and RB1. These lesions, especially in the liver, have lower immune cell infiltration, with different compositions, allowing a cancer permissive microenvironment. Initial findings show that MBC with a high tumor mutational burden correlated to shorter time to relapse among those with HR + /HER2− cancers^6^. A few predictive biomarkers have been validated in other important trials and can be useful, especially in later lines of therapy^7,8^. Precision medicine improves patient outcomes and quality of life with tailored treatments^8–10^. However, it remains a mystery why some patients relapse long after they have been considered cured, why some tumors grow quickly despite treatment while others respond exceptionally well for a long time to the same treatments. The answers are still out there as to what triggers some cancers to “change their spots”: to switch between different subtypes of breast cancer. In AURORA, this occurred in 36% of the tumors^6^.

## The AURORA program

To develop more tailored treatments for patients, we need to gain further insight into MBC. Most knowledge today relies on studies that used only primary tumor or metastatic biopsies taken after multiple lines of therapy. Cancers evolve and respond to biological pressures from systemic treatments. They are affected by the response of our immune system. Paired analysis of primary and the first metastatic relapse is an ideal way to investigate the disease in greater depth: what is different about the cancer that reappears after what should have been curative therapy? Most studies in the advanced setting have short follow-up, which may not be enough to capture patient’s exceptional response.

AURORA (NCT02102165), an initiative led by the Breast International Group (BIG, https://bigagainstbreastcancer.org/), has been performing comprehensive multi-omics analysis on matched primary and metastatic tumor samples as well as circulating tumor DNA (ctDNA) linked with curated clinical data from 1157 patients with metastatic disease. This fully academic program, primarily supported by the Breast Cancer Research Foundation (BCRF, https://www.bcrf.org/), started in 2014. Patients were included from >60 hospitals in 11 European countries and is the largest database of its kind so far.

AURORA aims to unravel the complexity of MBC and to provide new potential targets for drug development. Targeted next generation sequencing (NGS) technology is used to analyze primary and metastatic tumor biopsies taken prior to any systemic treatment and blood samples collected at baseline and during treatment or at disease progression. Patients are followed for up to 10 years to document baseline and further treatments, enrollment into clinical trials, and their clinical outcomes. Analysis of the first 381 patients has been published^6,11,12^.

The molecular profiling has two parts: a prospective and real-time analysis of DNA, and a retrospective and deeper analysis of RNA and genetic variants or copy number variation of the DNA. Results of the real-time analysis are reviewed and curated by a virtual Molecular Advisory Board (MAB) composed of expert biologists and clinicians and are provided back to clinicians for discussion with patients. Frozen metastatic tumor samples are stored at the AURORA biobank for future research. Pathology imaging scans and tumor-infiltrating lymphocyte scoring of all included patients are available.

**Fig. 1:**
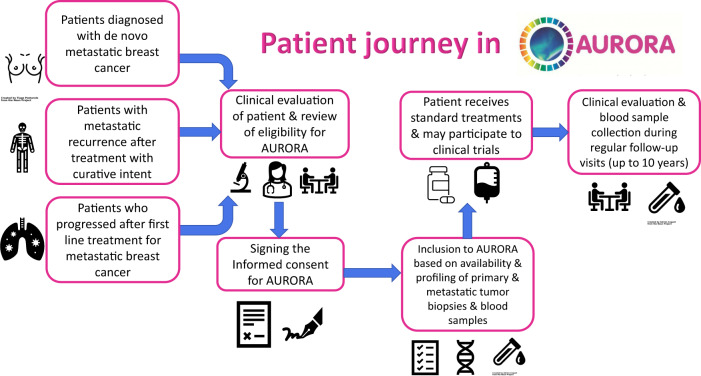
A patient’s journey in the AURORA program. This model of analyzing matched primary and metastatic samples coupled with data from prospective follow-up of patients is critical to a better understanding of metastatic breast cancer.

In AURORA, the full picture of a patient’s treatment journey can be linked with rich molecular data. This is critical to a better understanding of MBC (Fig. 1). It allows the comparison of tumor biology between at least two points: when the cancer is first diagnosed (the primary), and when it first reappears as incurable disease (metastasis). This is complemented by the liquid biopsies taken during treatment and at disease progression, and clinical information from multiple treatment lines linked with survival outcomes.

A defining feature of the program is the MAB, which curates the results of molecular profiling and identifies potential clinical trials into which the included patient may be enrolled. A MAB report specific to AURORA is generated for every patient. The choice of therapy remains at the discretion of the treating physician. Because these genomic results are generated in a research environment, the treating physician has to ensure that the results are confirmed using approved genetic tests prior to any clinical action. In many countries or territories, comprehensive NGS is not yet reimbursed for patients with early-stage or MBC^13,14^. AURORA provides valuable help in identifying patients who may be eligible for targeted treatment. Initial findings from the program showed that ESCAT tier I/II alterations were detected in 51% of patients but matched therapy was used only in 7%^6^. In terms of “clinician satisfaction”, an AURORA survey showed that 65% of recruiting physicians consider the MAB reports useful for their daily practice, although improvements are needed. This highlights the need to integrate multi-omics analyses into clinical practice to tailor treatment strategies, especially for vulnerable patients with metastatic disease.

## Transatlantic academic research collaboration

**Fig. 2:**
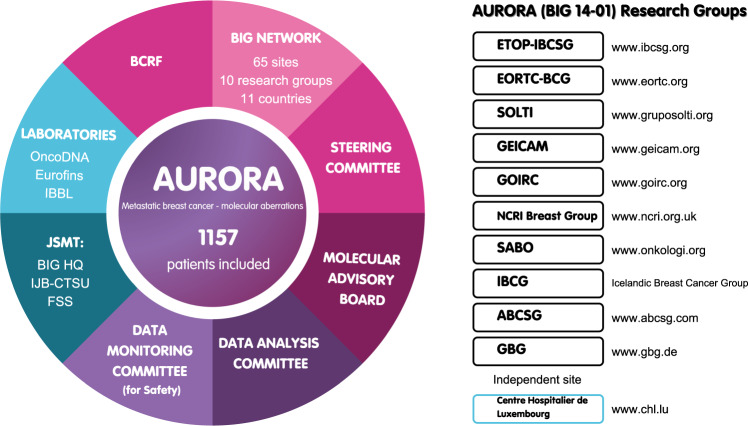
Key partners and committees involved in AURORA. Breast Cancer Research Foundation (BCRF), Breast International Group (BIG), Joint Study Management Team (JSMT), BIG Headquarters (BIG HQ), Institut Jules Bordet Clinical Trials Support Unit (IJB-CTSU), Frontier Science Scotland (FSS), Integrated Biobank of Luxembourg (IBBL), European Thoracic Oncology Platform-International Breast Cancer Study Group (ETOP-IBCSG), European Organization for Research and Treatment of Cancer Breast Cancer Group (EORTC BCG), Grupo Español De Estudio, Tratamiento Y Otras Estrategias Experimentales En Tumores Sólidos (GRUPO SOLTI), Grupo Español de Investigación en Cáncer de Mama (GEICAM), Gruppo Oncologico Italiano di Ricerca Clinica (GOIRC), National Cancer Research Institute (NCRI), Swedish Association of Breast Oncologists (SABO), Austrian Breast & Colorectal Cancer Study Group (ABCSG), German Breast Group (GBG).

Setting up AURORA back in 2014, overcoming all its challenges, and pushing forward towards completion of target recruitment in 2021 represents a triumph for BIG, its partners, and the academic community. The key partners are described in Fig. 2.

The program is independent of any pharmaceutical company. This is crucial for the academic community to identify the most relevant, patient-centered, and innovative research questions to be addressed^15^. However, an even stronger international collaboration is needed to harness the data and samples collected so far and to fund future meaningful research. We are open to collaborating with academic groups that have interest, knowledge, and funding to conduct in-depth analyses.

The European AURORA program (AURORA EU), with its counterpart AURORA US run by the Translational Breast Cancer Research Consortium (TBCRC), are both supported by the BCRF through the Evelyn H. Lauder Founder’s Fund for Metastatic Breast Cancer Research. AURORA was conceptualized and developed during the annual meetings between BIG and the National Clinical Trials Network (NCTN), which brings together some of the largest cooperative research groups in the world^16^. Since 2005, these initiatives have paved the way for clinicians and scientists to collaborate and address some of the most pressing challenges in breast cancer research. Both AURORA EU and US are working closely to overcome the hurdles of trans-Atlantic data-sharing and to advance science. Their mission is to deliver robust and high-quality data that will lead to improving the treatment and outcome of patients with MBC globally.

## Patient perspective on research priorities

Patients are at the heart of AURORA. Without their belief in the promise of precision medicine and their commitment to study procedures, AURORA would not be possible. As MBC is currently incurable, the patient perspective is essential to ensure relevant research questions are prioritized. There are aspects of a MBC journey that are more difficult for patients, most likely due to lack of the needed healthcare resource in a country, lack of coordination across institutions caring for cancer patients, or the stigma associated with metastatic disease. Some studies have shown the disparity in awareness and recognition of risk factors or symptoms between early-stage and MBC, the latter being less recognized by patients and even health care providers^17^. Research needs to consider the daily struggles and totality of patients, the real-life impact of interventions in this setting as much as their efficacy.

Within AURORA, patient advocates are part of the Steering Committee (SC), directly involved in the governance, scientific leadership, and communication of study results. The patient advocacy network Europa Donna is represented on AURORA’s SC and was involved early on in reviewing and improving the informed consent form, resulting in an easy to understand and relatively concise form commended by ethics committees and physicians. Patient advocates are the ambassadors of AURORA, bridging public awareness through in-person or virtual forums about MBC, and helping to generate much needed funds to keep the program going.

Beyond this, BIG is opening more possibilities for patients to be heard. With the aim to deepen its interaction with patients, to facilitate their involvement in defining research priorities, and contributing to early development of trials, BIG recently established its Patient Partnership Initiative. This is a core group of patient advocates who interact directly with BIG’s Executive Board as partners in developing and refining new trial concepts. As research moves forward, it is important to keep our anchor on what truly matters: patients. Their perspective is essential in defining priorities in this field and guiding decisions around the best use of data from molecular profiling programs like AURORA.

## Future of AURORA

**Fig. 3:**
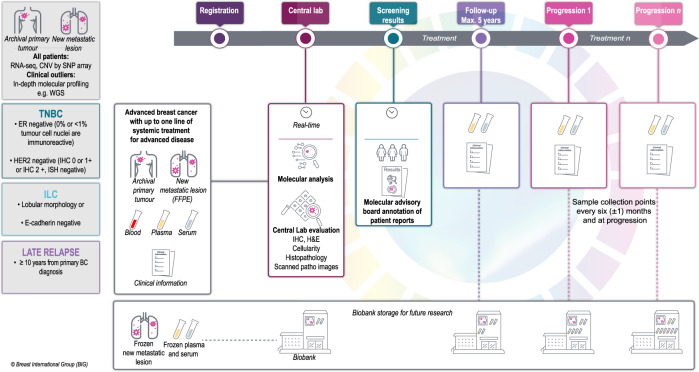
Study design of AURORA (Protocol version 4.0). Expansion to specific populations: patients with triple negative breast cancer (TNBC), invasive lobular breast cancer (ILC), and those whose disease relapsed >10 years after the primary diagnosis. RNA sequencing (RNA-Seq), Copy Number Variation (CNV), Single nucleotide polymorphism (SNP), Whole genome sequencing (WGS), Estrogen receptor (ER), Human epidermal growth factor receptor 2 (HER2), Immunohistochemistry (IHC), Breast cancer (BC), circulating tumor DNA (ctDNA), hematoxylin & eosin (H&E), formalin-fixed paraffin-embedded (FFPE).

MBC is the crucible of breast cancer research. Hence, the mission of AURORA continues. The next step is to expand the program to enrich the database with specific populations: patients with triple negative breast cancer, invasive lobular breast cancer, and those whose disease relapsed >10 years after the primary diagnosis (Fig. 3). These specific populations have been selected based on the high clinical unmet needs and the rapidly evolving research landscape. The target number of patients to be recruited is 250 over a period of 4 years, with follow-up set at 5 years. A more comprehensive diagnostic assay will be used to perform the molecular profiling of all included patients.

Following the analysis of its first 381 included patients, work is underway to perform a comprehensive analysis of 1157 included patients and to utilize >6000 available biological samples for innovative research. To produce an even more robust dataset on clinically relevant subgroups, AURORA EU and US are actively working together to share data and perform meaningful collaborative research. Data from the first AURORA manuscript are currently being shared through https://aurora.bigagainstbreastcancer.org/. Work is ongoing to improve this platform and share published data through the BCRF Global Data Hub (https://bcrfglobaldatahub.org/).

We acknowledge that AURORA has limitations. All patients have developed metastases, and hence no “control group” exists. We have paired samples, but we know about heterogeneity between metastatic sites. Because it is impossible to biopsy all sites, we count on ctDNA to capture some of this heterogeneity. To keep study costs in check, targeted NGS (amplicon based) was performed, not whole exome sequencing. Single cell RNA-Seq, DNA methylation sequencing or proteomic analyses were not performed. Nevertheless, the AURORA Core Data Analysis Committee has established robust methodologies to overcome these limitations and facilitate analyses.

## Conclusion

Conducting a fully academic international study like AURORA is challenging, yet ever more crucial to advancing knowledge about MBC. Our hope is that the findings from the AURORA EU and US initiatives will form the basis for well-designed and patient-centered clinical trials driven by precision medicine. The shared vision of all partners is that the program will lead to a deeper understanding of MBC and will contribute to trials that truly respond to patient’s needs. Stronger international collaboration across initiatives like AURORA EU and US gives hope to patients for whom time is never enough.

### Reporting summary

Further information on research design is available in the Nature Research Reporting Summary linked to this article.

## Supplementary information

Reporting Summary

## Data Availability

Data sharing is not applicable to this article as no datasets were generated or analyzed for it.
